# Two Incidental Sibling Diagnoses of Netherton Syndrome in Separate Visits: A Case Report

**DOI:** 10.7759/cureus.56439

**Published:** 2024-03-19

**Authors:** Samah AlMoosawi, Sara Alkhanaizi, Marwa Albaharna, Fatema Khamdan

**Affiliations:** 1 Dermatology, Salmaniya Medical Complex, Manama, BHR

**Keywords:** pityriasis rubra pilaris, psoriasis, icthyosiform erythroderma, atopic dermatitis, spink5 gene, netherton syndrome

## Abstract

Netherton syndrome (NTS) is a genetic disorder that predominantly affects the hair and the skin, and it can have a wide variety of presentations. The genetic syndrome is more common with consanguineous parents. Given the rarity and varying presentation of the condition, a few cases have been reported in the literature. We present an unusual case of two incidental diagnoses of NTS in siblings of consanguineous parents, manifesting as erythroderma and other symptoms that were initially diagnosed as pityriasis rubra pilaris and psoriasis in separate visits. Physicians must maintain a high index of suspicion when faced with chronic skin conditions and hair shaft abnormalities that may have been present since childhood to avoid the sequela of inadvertent prolonged or misdiagnosis.

## Introduction

Netherton syndrome (NTS) is an autosomal recessive disorder that can occur worldwide, with dominance in inbred populations [1]. Considering the phenotypic variability and clinical overlap with atopic dermatitis (AD) as well as other forms of ichthyosis, it has been estimated that the incidence of NTS might be as high as one in 50,000 [1]. It is characterized by the following cutaneous features: erythema, desquamation, generalized skin peeling, and pruritus. NTS is associated with hypotrichosis, trichorrhexis invaginata, and other hair shaft abnormalities. It is a result of mutations in the protease inhibitor gene SPINK5 [2]. A significant association has been observed between a particular SPINK5 variant, Glu420Lys, and AD, providing deeper insights into the pathogenesis of this complex disorder.

## Case presentation

**Figure 1:**
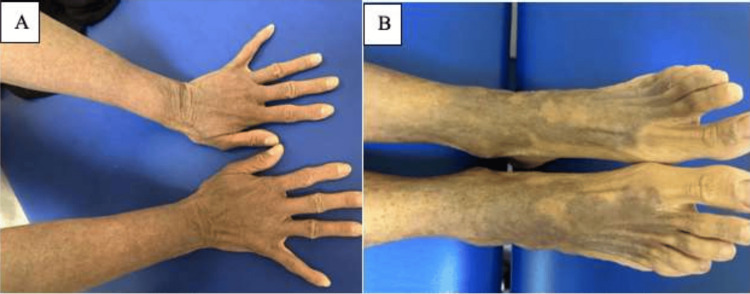
(A) Generalized xerosis and (B) bilateral lower limb ichthyosis (female patient)

A 58-year-old Bahraini woman presented to our dermatology clinic with a long-standing history of severe generalized xerosis and ichthyosis on both of her lower legs extending to the dorsum of her feet (Figure 1). She also had extensive erythema with peeling over the neck, face, axilla, chest, thighs, and legs with islands of sparing. She complained of fragile hair, reporting that it has been short since childhood. She was initially diagnosed with psoriasis and given topical steroids. A skin biopsy was taken by the patient’s previous dermatologist which was suggestive of pityriasis rubra pilaris (PRP). Accordingly, she was started on topical phototherapy with adjunctive topical steroids. The patient completed a total of 24 sessions of phototherapy treatment (narrow-band ultraviolet B, 3007 mJ) and reported improvement in overall symptoms.

**Figure 2:**
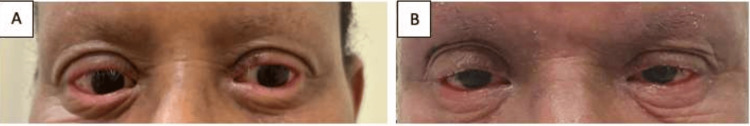
Bilateral ectropion (A) Female patient. (B) Male patient

After further exploration of the patient’s family history, it was revealed that her parents are consanguineous and that her son has AD. She reported that her late mother had similar symptoms, and her brother currently suffers from the same symptoms. On examination, the patient had severe generalized xerosis, pili torti, and trichorrhexis nodosa, which was confirmed upon trichoscopy. An eye examination showed bilateral ectropion (Figure 2), loss of the outer third of the eyebrows, and the absence of eyelashes.

**Figure 3:**
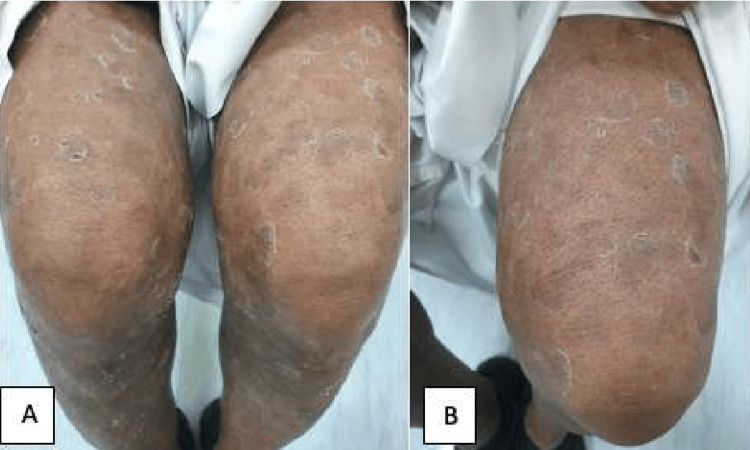
(A) Generalized involvement with features of congenital ichthyosiform erythroderma. (B) Close-up demonstrating the “peeling” quality of the scales (male patient)

**Figure 4:**
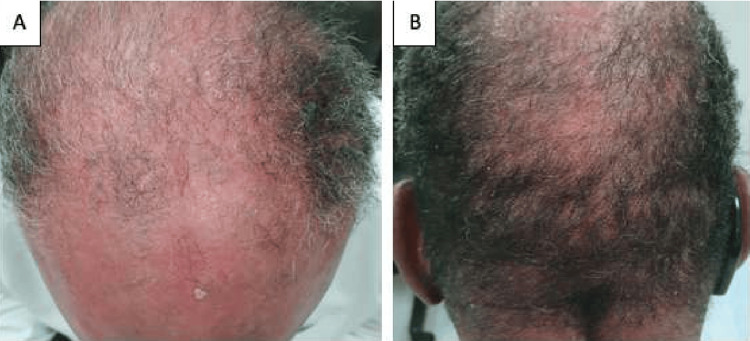
(A) Short, thin hair on the scalp with areas of baldness. (B) Sparse hair distribution

The following week, a 62-year-old male patient presented to our dermatology clinic with multiple peeling, erythematous scales, and plaques over his lower limbs and trunk (Figure 3). His condition was initially diagnosed in a different tertiary hospital as psoriasis, and he was started on topical treatment. However, because of his poor response and severity of skin lesions, he was shifted to weekly adalimumab injections, which did not bring out symptomatic relief either. The patient then developed generalized thickening of the skin, erythroderma, and exfoliating dermatoses with the sparing of his palms and soles. Hair findings included short, brittle, and grayish hair (Figure 4) trichorrhexis nodosa and pili torti were evident upon trichoscopy. Similar to his sister, an eye examination showed bilateral ectropion. Consequently, biological treatment was stopped, and a skin biopsy was taken. The histopathology report was suggestive of erythrokeratodermia variabilis (NTS), and the patient was referred to genetics accordingly. Upon his follow-up appointment, he presented with extensive scaling of the scalp and ichthyosiform rash all over his body. The erythroderma was resolved, but lower limb desquamation was persistent. The patient was offered to start a trial of Neotigason (acitretin) or weekly phototherapy sessions. 

Genetics

Both siblings had genetic testing done, with the result showing the presence of gene mutation, SPINK5 (+) (ENST00000256084.8) Exon 26 c.2471_2474del (p.Lys824ArgfsTer99) homozygous, classified as pathogenic mutation, confirming the diagnosis of NTS (autosomal recessive).

## Discussion

In many patients, NTS presents at or soon after birth with scaling and generalized erythroderma or with ongoing peeling of the skin [3]. In patients with severe disease, both generalized ichthyosis and erythroderma persist throughout life [3]. Over time, the distribution of the plaques varies in shape, size, and location on the trunk and extremities, proving the surge in NTS. Because of the pruritic nature of the lesions, some patients are predisposed to lichenification and the development of eczematous plaques on flexures of the skin [3]. In terms of the scalp, thick scales are commonly observed in NTS. Abnormalities of the hair shaft tend to improve with age. However, there is a wide disparity in extent, age of onset, type, and severity [3].

Hair shaft abnormalities in NTS include trichorrhexis invaginata (bamboo hair). Additionally, trichorrhexis nodosa, pili torti, and helical hair may be observed. Eyelashes and secondary sexual hair can be affected as well [3]. Another characteristic feature of NTS is immune dysregulation, which includes atopic manifestations and immunodeficiency involving B cells and natural killer cells. When observing laboratory work-up, there is a marked increase in the serum levels of IgE, ranging from 100 to 10,000 IU/mL. It is common to find eosinophilia and allergic reactions to foods or antigens, which may manifest as urticaria, angioedema, or in severe cases, anaphylactic shock [4].

Failure to thrive is often noted in patients with generalized skin involvement, which may be associated with an enteropathy and the high caloric needs related to developing erythroderma, which subsequently leads to short stature [4]. Moreover, sporadic aminoaciduria is another consequence of the generalized skin involvement, which may affect the normal development of the patient [5].

A pathognomonic feature of NTS is usually trichorrhexis invaginata; however, its appearance is usually delayed. Therefore, inspection of the hair should be prioritized at all stages [6]. In families with known SPINK5 mutations, prenatal diagnosis can be done using chorionic villus sampling (CVS) or amniocentesis material [2]. Additionally, skin biopsy specimens may be immunostained with anti-LEKT1 antibodies which determine the absence or irregular dissemination of LEKT1 protein in the epidermis [7]. While staining of skin biopsy via immunohistochemistry has been suggested as a useful diagnostic test for NTS, identification of the disease-causing mutation via genetic testing is ideal for confirmation of the diagnosis [8].

In terms of differential diagnoses, peeling skin syndromes (PSSs) can be considered NTS. They are a heterogeneous group of rare, autosomal recessive disorders characterized by superficial, painless peeling, and blistering of the skin without mucosal fragility [9]. The shared similarity with NTS includes congenital erythroderma, histologic, and ultrastructural malformations [3]. Moreover, corneodesmosin gene (CDSN) mutations may also cause generalized inflammatory PSS [10]. The differential diagnoses may include other erythrodermas in the infantile category, specifically non-bullous congenital ichthyosiform erythroderma, and erythrodermic psoriasis [8]. Exclusion of other conditions such as AD, lamellar ichthyosis, primary immunodeficiency syndromes, seborrheic dermatitis, and acrodermatitis enteropathica is necessitated [8].

The mainstay treatment for NTS is symptomatic relief, which is catered to the patient’s specific needs [3]. It is important to note that symptoms of NTS can become less apparent with age. Periods of little or no disease symptoms are interspersed with sporadic aggravations [11]. The ideal treatment modality includes topical emollients, keratolytics, tretinoin, calcipotriol, and corticosteroids alone or in combination [3]. For instance, if pruritus is one of the main complaints, initiation of oral antihistamines in NTS can help provide symptomatic relief. However, since patients who suffer from NTS experience severe skin barrier dysfunction, the skin lesions that are treated with topical tacrolimus can be at risk of increased percutaneous absorption [12]. Thus, upon initiation of topical tacrolimus, monitoring of plasma drug level is of utmost importance [3]. An alternative treatment option could be topical pimecrolimus as it was proven to be successful with less systemic absorption and, thus, fewer side effects [13]. It has also been reported that patients may highly improve on treatment with narrowband ultraviolet B (UVB), psoralen plus ultraviolet A (PUVA), ultraviolet A1 (UVA1), and balneophototherapy (broadband UVB plus saltwater baths) [3].

In the medical literature, there are approximately 150 cases of NTS that have been reported, but the true number of individuals that are affected may be much higher because of diagnostic challenges and overlapping symptoms with other congenital ichthyoses [14].

## Conclusions

Recognizing NTS promptly can be challenging for dermatologists because of the complexity of the genetic syndrome and its expansive variation of clinical presentations. A high index of suspicion must be maintained when faced with chronic ichthyosiform erythroderma and hair shaft abnormalities that may have been present since childhood. Considering the limited data in the literature, the ideal management must be decided and tailored according to individualistic clinical presentation and symptom severity. Although both patients were seen by qualified, board-certified dermatologists in tertiary hospital settings, the ambiguous presentations of NTS have led to an inadvertent deferral in confirmation of the diagnosis for both siblings, causing an increase in the interval between the appearance of symptoms and their management. Further comprehension of the syndrome and research into its underlying features is warranted to widen the knowledge of physicians on NTS.
